# Electrically controlled terahertz magneto-optical phenomena in continuous and patterned graphene

**DOI:** 10.1038/ncomms14626

**Published:** 2017-03-07

**Authors:** Jean-Marie Poumirol, Peter Q. Liu, Tetiana M. Slipchenko, Alexey Y. Nikitin, Luis Martin-Moreno, Jérôme Faist, Alexey B. Kuzmenko

**Affiliations:** 1 Department of Quantum Matter Physics, University of Geneva, Geneva 4 CH-1211, Switzerland; 2 Institute for Quantum Electronics, Department of Physics, ETH Zurich, Zurich CH-8093, Switzerland; 3 Instituto de Ciencia de Materiales de Aragon and Departamento de Fisica de la Materia Condensada, CSIC-Universidad de Zaragoza, Zaragoza E-50009, Spain; 4 CIC nanoGUNE, Donostia-San Sebastián E-20018, Spain; 5 IKERBASQUE, Basque Foundation for Science, Bilbao 48013, Spain

## Abstract

The magnetic circular dichroism and the Faraday rotation are the fundamental phenomena of great practical importance arising from the breaking of the time reversal symmetry by a magnetic field. In most materials, the strength and the sign of these effects can be only controlled by the field value and its orientation. Furthermore, the terahertz range is lacking materials having the ability to affect the polarization state of the light in a non-reciprocal manner. Here we demonstrate, using broadband terahertz magneto-electro-optical spectroscopy, that in graphene both the magnetic circular dichroism and the Faraday rotation can be modulated in intensity, tuned in frequency and, importantly, inverted using only electrostatic doping at a fixed magnetic field. In addition, we observe strong magneto-plasmonic resonances in a patterned array of graphene antidots, which potentially allows exploiting these magneto-optical phenomena in a broad THz range.

## Abstract (web summary)

Time reversal symmetry breaking gives rise to magnetic circular dichroism and Faraday rotation in graphene. The authors use terahertz magneto-electro-optical spectroscopy to demonstrate that electrostatic doping at a fixed magnetic field allows inversion of magnetic circular dichroism and Faraday rotation.

Terahertz waves are actively explored for fundamental research and applications^1^,^2^. However, there is a lack of materials and technology allowing the fabrication of efficient and broadly tunable passive terahertz devices. Graphene, where the doped charge carriers interact strongly with the terahertz radiation^3^,^4^,^5^,^6^,^7^,^8^,^9^ and the doping is electrostatically tunable^10^, is promising to tackle this problem^11^,^12^,^13^. Moreover, graphene–light interaction is greatly enhanced if graphene plasmons^14^,^15^,^16^,^17^,^18^,^19^,^20^,^21^,^22^ are excited by patterning^4^,^23^,^24^. An additional advantage of graphene is the small cyclotron mass making the Drude response highly sensitive to the magnetic field^7^,^8^. While the mass is also small in conventional two-dimensional electron gases (2DEGs)^25^, the fundamental difference of graphene is that the charge carriers are Dirac fermions, where the cyclotron mass depends on doping^26^, and that the ambipolar gating is possible^10^. Moreover, while cryogenic temperatures are needed to observe magneto-optical (MO) effects in 2DEGs, in graphene they show up already at room temperature^27^. Unfortunately, not all the mentioned graphene benefits were explored so far in a single experiment.

Here we combine MO THz spectroscopy with electrostatic gating and graphene patterning. This allows us to observe several new phenomena, including an electrostatic control of the THz cyclotron frequency, a purely electrostatic inversion of the magnetic circular dichroism (MCD) and the Faraday rotation (FR) and strong gate-tunable magneto-plasmonic resonances in periodic antidot arrays. We argue that these effects potentially allow a broad spectral tunability and enable novel functionalities in graphene-based passive terahertz devices.

## Results

### Continuous graphene

**Figure 1:**
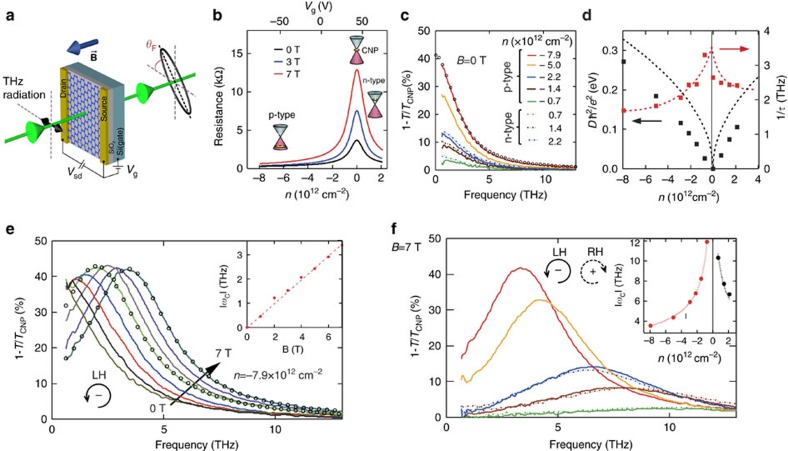
Doping-dependent magneto-resistance and magneto-optical extinction spectra of continuous graphene. (**a**) Schematic representation of a terahertz g-FET device and the optical experiment. (**b**) Two-terminal source-drain resistance at *B*=0, 3 and 7 T as a function of the gate-induced doping. (**c**) Extinction spectra at *B*=0 T for various doping levels and polarities. The open circles represent the best Drude fit for *n*=−7.9 × 10^12^ cm^−2^. (**d**) Doping dependence of the Drude weight relative to the CNP (black symbols) and the scattering rate (red symbols). The black line is the theoretical prediction for Drude weight of Dirac fermions for *v*_F_=10^6^ m s^−1^. The red line is the guide to the eye. (**e**) The extinction (for the LH circular polarization) at *n*=−7.9 × 10^12^ cm^−2^ for different values of magnetic field from 0 to 7 T with the step of 1 T. The open circles represent the best Drude fits at 4 and 7 T. The inset presents the field dependence of the CR frequency (circles) and the linear fit (dashed line). (**f**) The extinction spectra at 7 T as a function of doping. The colour legend is the same as in **c**. The spectra are shown for the LH/RH circular polarizations for the p-/n-doped regimes respectively, that is, for polarizations exhibiting the cyclotron resonance. The inset presents the doping dependence of the experimental cyclotron frequency (circles) and a fit using a Dirac-fermion model as described in the text (dashed lines). All measurements are done at *T*=250 K.

We first discuss the intrinsic MO terahertz effects in continuous (unpatterned) graphene. A basic device used in our experiments is a large-area CVD (chemical vapour deposition)-graphene field-effect transistor (g-FET) sketched in Fig. 1a. It allowed us to combine ambipolar control of doping *n* (positive for electrons and negative for holes) with magneto-transport and MO measurements in a perpendicular magnetic field *B* (positive towards the light source). The THz transmission *T*(*ω*) and the FR spectra *θ*_F_(*ω*) were obtained while graphene was illuminated by a linearly polarized light with the angular frequency *ω*. The Fabry–Perot interference in the substrate was suppressed intentionally to simplify the spectra analysis. From these measurements, we extracted the transmission spectra for the right-hand (RH)/left-hand (LH) circular polarizations, *T*_±_(*ω*)^28^, which fully characterizes the MO response of the device.

The source-drain resistance as a function of the gate voltage *V*_g_ in all our devices has a characteristic peaked shape (Fig. 1b) with the charge neutrality point (CNP) at a positive voltage, indicating a residual p-type doping. The curves show a strong positive magnetoresistance and are electron–hole symmetric, demonstrating the high quality of our samples. The charge-carrier mobility *μ*≈3,500 cm^2^ V^−1^ s^−1^ and the doping inhomogeneity *δn*≈5 × 10^11^ cm^−2^ are deduced using a standard analysis of the transport curves^29^. Figure 1c shows the zero-field extinction spectra 1−*T*(*ω*)/*T*_CNP_(*ω*), which are representative of the optical absorption in graphene (as specified in Supplementary Note 2 and shown in Supplementary Fig. 2). The strong exctinction increase at low frequencies is due to the Drude absorption by the doped charges^3^,^4^,^5^,^6^,^9^. The fact that almost half of terahertz photons are stopped by gate-injected carriers in an atomic monolayer demonstrates the remarkable efficiency of the Drude response. Importantly, the extinction curves at the matching p- and n-type doping levels are similar, which is consistent with the symmetric shape of the transport curves.

Optical conductivity is described almost perfectly (as shown by the open circles in Fig. 1c) using a semiclassical Drude model:


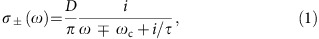


where *D* is the Drude weight, *ω*_c_ is the cyclotron frequency (positive/negative for n-/p-type doping), *τ* is the scattering time and ± refer to RH/LH circular polarizations. A relation between *σ*_±_ and *T*_±_ that takes the substrate into account is given in Supplementary Note 1 and illustrated in Supplementary Fig. 1.

Before we analyse the dependence of the Drude parameters on the doping and the magnetic field, we stress that the Dirac-fermion theory (in the semiclassical limit) predicts^26^ that


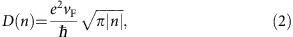


and


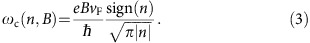


where *v*_F_ is the Fermi velocity, *ħ* the reduced Planck's constant and *e* the (positive) elementary charge. This behaviour is different from the case of conventional 2DEGs^25^, where the Drude weight is proportional to *n*, while the cyclotron frequency is *n*-independent. Notably, equation (3) was not tested so far since in all terahertz spectroscopic measurements the electrostatic gating was done separately from applying a magnetic field.

The Drude weight and the scattering rate 1/*τ* extracted by fitting the zero-field extinction curves (where *ω*_c_=0) are strongly doping dependent (Fig. 1d). A theoretical dependence (dashed line) for a typical value of *v*_F_=10^6^ m s^−1^ agrees reasonably well with our experiment, with the experimental values being about 20% lower, in a qualitative agreement with a previous report^3^. We did not observe any significant change of the Drude weight with the magnetic field. As observed in a recent report^9^, the scattering rate decreases as the doping increases for both charge polarities. This effect, which is beneficial for MO applications, is consistent with either a doping-induced screening of charged impurities^30^,^31^,^32^ or with the resonant scattering^33^.

In a magnetic field, the terahertz spectra are dominated by the cyclotron resonance (CR) (Fig. 1e,f). The extinction curves for a strongly p-type-doped sample (*n*=−7.9 × 10^12^ cm^−2^) for the LH circular polarization, where the CR is observed in this case, are shown in Fig. 1e for different magnetic field values up to 7 T. The Drude fit works equally well in magnetic field, as shown by open circles for 4 and 7 T. The cyclotron frequency shifts linearly with *B* (inset of Fig. 1e) and a record extinction of 43% is reached at 5 T at a frequency as high as 2.5 THz. Furthermore, Fig. 1f reveals that the cyclotron frequency strongly increases (up to 10 THz) with the reduction of the charge concentration for both doping regimes. This is a spectacular fingerprint of Dirac fermions, as theory indeed predicts *ω*_c_ to be doping dependent, according to equation (3). This relation fits the experimental data very well (inset of Fig. 1f), where similar Fermi velocity values for p- and n-type doping regimes are found (1.01±0.02 and 0.92±0.05 × 10^6^ m s^−1^, respectively).

**Figure 2:**
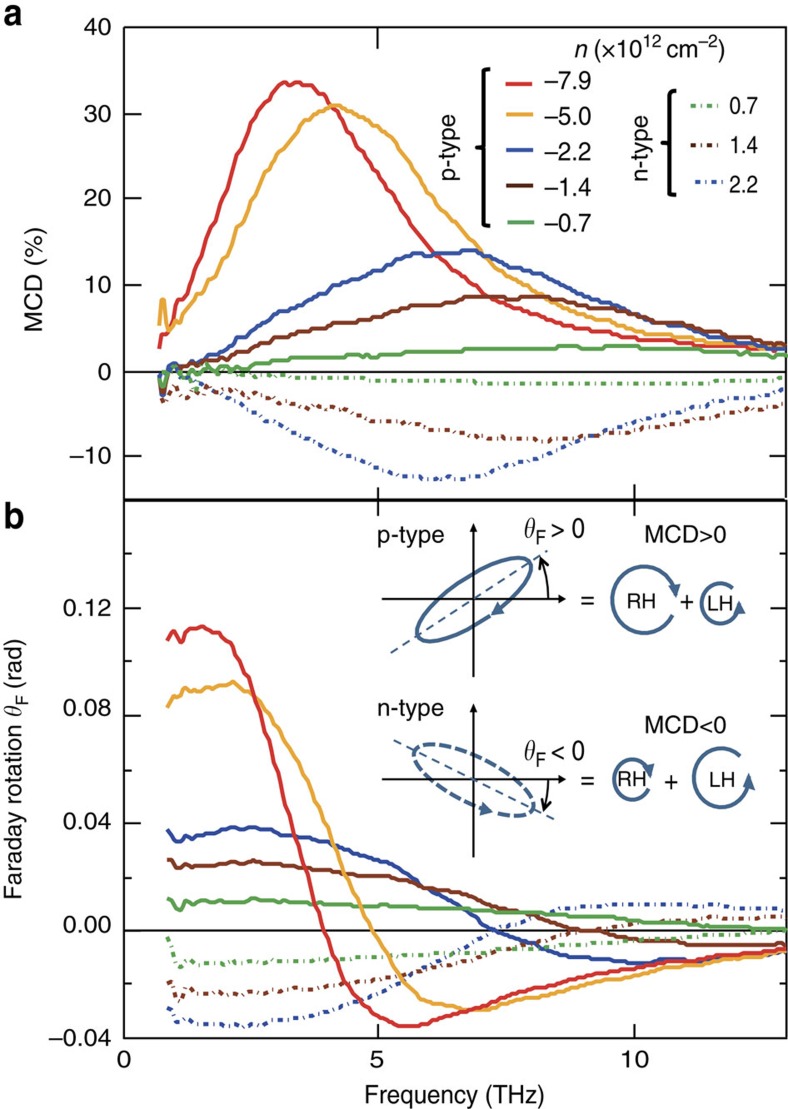
Electrostatic sign inversion of magnetic circular dichroism and Faraday rotation in continuous graphene. (**a**,**b**) Spectra of MCD=(*T*_−_−*T*_+_)/*T*_CNP_ and FR *θ*_F_ at different doping levels at 7 T. The inset compares the polarization ellipses of the transmitted radiation and illustrates the opposite signs of the MCD and the FR for the p- and n-type doping. All measurements are done at *T*=250 K.

Figure 2 shows the MCD (defined as the difference between the extinction coefficients for the RH and LH circular polarizations) and the FR spectra at 7 T for different doping values. Both quantities can be non-zero only if the time reversal symmetry is broken and are the key parameters for the non-reciprocal light manipulation. We note that the high values of MCD and FR (in the present case, 35% and 0.11 rad, respectively) demonstrate the potential of graphene as a MO material in this frequency range. Furthermore, Fig. 2 reveals that in addition to tuning the frequency and modulating the intensity of the magneto-extinction, the electrostatic doping also allows the inversion of the magneto-circular dichroism and the FR at a fixed magnetic field. Indeed, in both cases the spectra corresponding to equal absolute carrier concentrations but opposite doping types show a high degree of symmetry with respect to zero. This fully agrees with the Dirac-fermion theory predicting the inversion of the cyclotron frequency (equation (3)) at a constant Drude weight (equation (2)) as *n* changes sign. The inversion of the MCD is thus due to the fact that the polarization where the CR is observed is defined by the doping type. This new effect that enables novel applications (as discussed below) is thus directly related to the unique possibility of ambipolar doping control in graphene^10^.

### Patterned graphene

**Figure 3:**
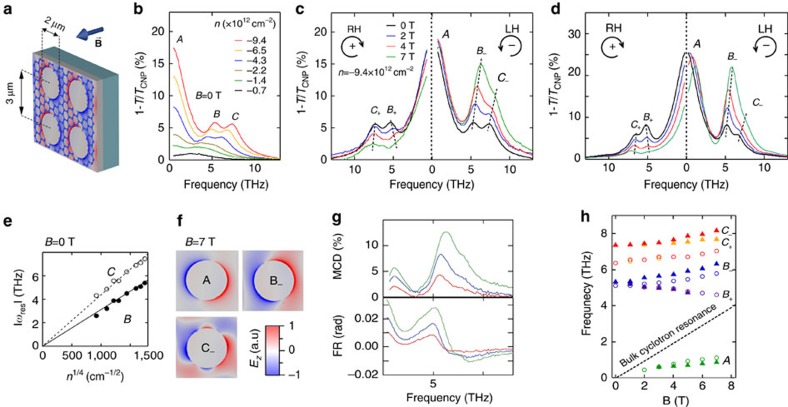
Electro-magneto-optical terahertz experiment and simulations in antidot-patterned graphene. (**a**) Schematic illustration of the patterned antidot array. Electrical contacts and terahertz light are the same as in Fig. 1a and are not shown. The superimposed colour image exemplifies a distribution of the perpendicular AC electric field, when a magneto-plasmon mode (*B*_−_) is excited. (**b**) Experimental extinction spectra at *B*=0 T for various carrier concentrations (p-doping). Symbols *A*, *B* and *C* indicate the resonant peaks. (**c**) Experimental MO extinction spectra for RH and LH polarizations at *n*=−9.3 × 10^12^ cm^−2^ at the selected magnetic fields. The black dashed lines are guides to the eye to follow the evolution of peaks *B* and *C*. (**d**) Finite-element simulation of the experimental data at the same doping level and the magnetic field values as in **c**. As in **c**, the black dashed lines follow peaks *B* and *C*. (**e**) Doping dependence of the Bragg peaks *B* and *C* extracted from **b**. (**f**) Distribution of AC electric field *E*_*z*_ corresponding to the modes *A*_−_, *B*_−_ and *C*_−_ at 7 T as shown in **d**. (**g**) Spectra of MCD and FR corresponding to **c**. (**h**) Magnetic field dependence of experimental (solid triangles) and simulated (open circles) magneto-plasmon frequencies corresponding to **c**,**d**. The black dashed line indicates the bulk cyclotron resonance *ω*_c_(*B*). All measurements are done at *T*=250 K.

As demonstrated above, the non-reciprocal MO effects in unpatterned graphene are especially strong at low frequencies (below 5–7 THz). However, in the higher THz region the Drude response is less efficient. In order to broaden further the tuning range of the magneto-teraherz response, one can use the new physical properties arising from the excitation of magneto-plasmons (that is, collective charge oscillations in external magnetic field) in graphene nanostructures^23^,^34^,^35^,^36^,^37^. To this end, we patterned graphene with a periodic antidot array (Fig. 3a) that acts as a two-dimensional plasmonic crystal^24^. The transport curves (presented in Supplementary Fig. 3) are similar to the ones in continuous graphene (Fig. 1b), indicating that patterning does not strongly degrade the charge mobility. In order to better understand the magnetic field dependence, we first show in Fig. 3b the zero-field extinction spectra of patterned graphene with a period of 3 μm and a hole diameter of 2 μm that were studied previously by us^24^. They feature a Drude-like peak (*A*) as in continuous graphene and two additional peaks (*B* and *C*) due to Bragg scattering of graphene plasmons on the periodic structure. Our previous study revealed that peak *B* is mostly due to the (0, 1)-order Bragg reflection, while peak *C* has a mixed (1, 1)–(0, 2) character. Importantly, they both demonstrate a 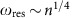 dependence typical for Dirac fermions (Fig. 3e)^4^,^14^,^15^ that allows controlling the resonance frequency by the gate voltage, in addition to tuning it by the antidot dimensions and spacing^24^.

A comparison of Figs 1c and 3b reveals that the extinction in patterned graphene is in fact lower than or comparable to the extinction in continuous graphene at the same frequencies, even close to plasmonic resonances. A certain reduction of the extinction is because of the removal of graphene by patterning and the corresponding reduction of the filling factor (about 40% in our case). Second, this is a consequence of a relatively low mobility, which broadens and reduces both the plasmon and the Drude peaks. Therefore, in patterned samples with a high enough mobility, where the peaks are expected to be sharper and higher, the extinction should be higher than in continuous graphene.

The magnetic field affects strongly the extinction curves (Fig. 3c) and shifts the peak frequencies (solid symbols in Fig. 3h). Peak *A* shifts upwards in the LH polarization while keeping the intensity constant. Although this resembles the behaviour of the CR peak in continuous graphene (Fig. 1e), the frequency of peak *A* is much lower than the bulk CR for the same values of *n* and *B* and shows a sublinear field dependence. A similar excitation was observed in 2DEGs^38^ and identified as a cyclotron mode at low magnetic fields crossing over to a so-called edge magneto-plasmon circulating around the holes as the field is increased. Bragg magneto-plasmon peaks *B* and *C* exhibit a dramatic field dependence and a totally different behaviour for the RH and LH circular polarizations (where we denote them *B*_+_, *C*_+_ and *B*_−_, *C*_−_ respectively). Most notable is a strong increase/decrease of their intensity for the LH/RH polarization, accompanied by field-induced shifts as indicated by the dashed lines in Fig. 3c. The polarization frequency splitting is most prominent for peak *B*, where opposite shifts are observed in the two polarizations. Associated with these spectacular magneto-plasmonic effects are the resonant MCD and FR structures (Fig. 3g), which are present at much higher frequencies than resonances in continuous graphene (Fig. 2), confirming that patterning indeed extends significantly the spectral range of the strong MO activity.

These results agree qualitatively with low-temperature experiments^38^,^39^,^40^ and theory^41^,^42^ in conventional 2DEG antidot arrays. However, it is imperative to quantitatively understand the present spectra in order to predict the performance of graphene THz devices. To this end, we performed a finite-element electromagnetic simulation of our system, where the Drude weight *D* and the cyclotron frequency *ω*_c_ were determined using equations (2) and (3), while the scattering time *τ* was set to 0.1 ps in order to match the observed width of the magneto-plasmon peaks. The simulated extinction spectra for various magnetic field values are shown in Fig. 3d and the corresponding mode frequencies are shown as open circles in Fig. 3h One can notice an excellent agreement with the experimental curves (Fig. 3c), in terms of the spectral shapes and the evolution of the peak positions and intensities as a function of *B*. In particular, the simulation reproduces perfectly the sublinear field dependence of peak *A* and the opposite field-induced shifts of the *B*_−_ and *B*_+_ peaks.

To give a further physics insight into the origin of the observed MO resonances, we show in Fig. 3f the simulated distributions of the AC electric field *E*_*z*_ perpendicular to the sample for the *A*, *B*_−_ and *C*_−_ modes at 7 T. The electric field for the mode *A* is strongly localized near the antidot edge, corroborating its prior identification as an edge magneto-plasmon^40^,^41^,^42^ where the cyclotron orbits skip around the antidots. In contrast, the Bragg modes *B*_−_ and *C*_−_ are characterized by peculiar field distributions in the entire unit cell. At the edge, mode *B*_−_ has two *E*_*z*_ nodes that arise from the coupling between the dipolar plasmon and the CR. Mode *C*_−_ exhibits six nodes, originating from the mentioned above mixed-order Bragg reflection character. Because of this, mode *C* may be more sensitive to a possible variation of the graphene doping near the edge, not taken into account in the simulation, which could be the reason for some discrepancy (about 1 THz) between the experimental and the simulated frequencies. Nevertheless, overall our simulations based on the Dirac-fermion theory prove to be quantitatively accurate also in patterned graphene.

## Discussion

Our results indicate that combining terahertz spectroscopy, electrostatic gating, magnetic field and pattering reveals interesting properties of the Dirac fermions, such as a broadband electrostatic control of the cyclotron frequency and relativistic magneto-plasmons, and give rise to qualitatively new phenomena, such as a purely electrostatic control of the magnetic effects (MCD and FR). Importantly, the experiments were done close to room temperature, in a striking contrast to the existing 2DEG studies^38^,^39^,^40^, where the samples have to be cooled <4 K. This suggests the fundamental possibility of graphene-based terahertz non-reciprocal devices exploiting these effects. Examples of such devices are optical modulators, polarization converters, isolators and circulators, which are essential elements lacking in the present and future terahertz applications (life sciences, material characterization, telecommunications, homeland security and so on). As the strength of MO effects depends critically on the charge mobility, improving this parameter is essential to achieve a higher MO performance. The value of *μ*≈3,500 cm^2^ V^−1^ s^−1^ in our present g-FETs is largely limited by the substrate (SiO_2_/Si), and high magnetic fields (up to 7 T) are needed to attain reasonably good device specifications^43^. On the other hand, much higher mobility values^44^ and much longer optical scattering times^45^,^46^ can be achieved in graphene on boron nitride (BN). Notably, a mobility of ∼100,000 cm^2^ V^−1^ s^−1^ at room temperature was recently obtained in h-BN-encapsulated CVD graphene using a dry-transfer technique^47^. Although at present the small size of graphene–BN samples make MO THz experiments difficult, using CVD technique in combination with h-BN encapsulation^47^ is highly promising for future large-scale applications of high-mobility graphene. This would allow using cheap permanent magnets making the teragertz MO applications commercially relevant.

As we demonstrated, the unusual properties of Dirac fermions allow a wide tuning of the working (cyclotron or magneto-plasmon) frequency, potentially in the entire THz range, by the doping, magnetic field and patterning parameters. Moreover, the possibility to invert the MCD and the FR electrically opens the door to completely new functionalities, such as electrically switchable isolators, and to combining several basic functions in a single device.

Importantly, in the present case the MO effects occur in an atomically thin layer, therefore the device thickness is limited only by the substrate and external optical elements (polarizers, waveplates, magnets). This potentially allows a miniaturization of the graphene-based elements, in a stark contrast with conventional ones, where light has to travel a centimetre-scale distance. The scope of the MO terahertz applications of graphene is certainly not limited to free-space wave propagation, as in the present experiments, and we anticipate that its remarkable properties will be employed in miniaturized chip- and THz waveguide-integrated^48^ devices.

## Methods

### Experiment

Large-area monolayer CVD graphene was grown on copper and transferred to a weakly doped Si substrate with a 300 nm layer of SiO_2_ that served as a back-gate dielectric. Au/Ti electrodes were deposited outside the illuminated region (5 × 5 mm^2^) allowing transport measurements and back-gate control simultaneously with MO studies. The carrier concentration was calculated using the formula *n*=*α*(*V*_g_−*V*_g, CNP_), where *α*=7.2 × 10^10^ cm^−2^ V^−1^ is the gate capacitance and *V*_g, CNP_ is the gate voltage corresponding to the maximum resistivity. A perpendicular magnetic field up to 7 T was applied using a superconducting split-coil system. The sample was illuminated by linearly polarized light and the terahertz transmission and FR spectra using the technique presented in ref. 28 were measured close to the room temperature (250 K) as a function of the carrier concentration and magnetic field with the help of a Fourier transform spectrometer equipped with a bolometer detector. In order to simplify the data analysis, the Fabry–Perot interference in the Si substrate (0.5 mm thick in our samples) was suppressed by reducing the optical resolution to 0.2 THz. By applying MO Kramers–Kronig analysis^28^ to these data (shown in Supplementary Fig. 4), we derived the transmission spectra for RH and LH circular polarizations without the need of physically generating circularly polarized waves.

### Simulations

Finite-element electromagnetic simulations of antidot arrays were performed using the COMSOL software. The graphene layer was modelled as a surface current in the boundary conditions. In order to achieve convergence, the mesh element size in the vicinity of graphene was much smaller than the magneto-plasmon wavelength.

### Data availability

The data that support the findings of this study are available from the corresponding author upon request.

## Additional information

**How to cite this article:** Poumirol, J.-M. *et al*. Electrically controlled terahertz magneto-optical phenomena in continuous and patterned graphene. *Nat. Commun.* **8,** 14626 doi: 10.1038/ncomms14626 (2017).

**Publisher's note**: Springer Nature remains neutral with regard to jurisdictional claims in published maps and institutional affiliations.

## Supplementary Material

Supplementary Information Supplementary Figures, Supplementary Notes and Supplementary References
