# Supplementary material for: Electrically controlled terahertz magneto-optical phenomena in continuous and patterned graphene
Source: Nat Commun. 2017 Mar 7;8:14626. doi: 10.1038/ncomms14626 (PMC5344301; doi:10.1038/ncomms14626)
Supplement: Supplementary Information — Supplementary Figures, Supplementary Notes and Supplementary References [file ncomms14626-s1.pdf]

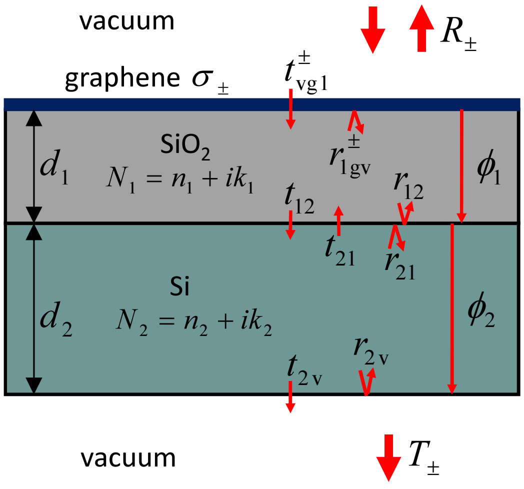

Supplementary Figure 1. Magneto-optical model of g-FET and description of the Fresnel coefficients used to compute the total magneto-optical transmission.

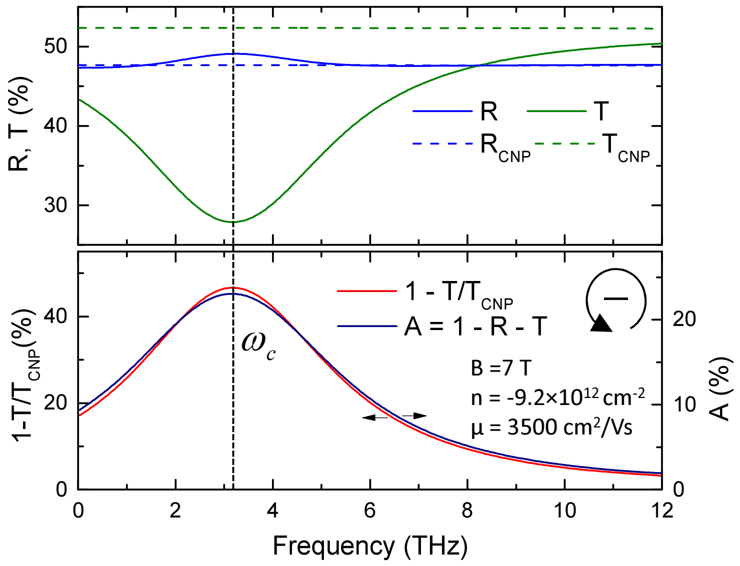

Supplementary Figure 2. Top panel: model spectra of reflectivity (green), transmission (blue) of a g-FET at B = 7 T with $n = -9.2 \times 10^{12}$ cm^{-2} (solid lines) and at the charge-neutrality point (dashed lines): The mobility μ=3,500 cm^{2}V^{-1}s^{-1}, which is close to the experimental value. Bottom panel: the corresponding spectra of extinction $1 - T/T_{\rm CNP}$ and absorption $A = 1 - R - T$.

I. SUPPLEMENTARY NOTE 1: MODEL FOR MAGNETO-OPTICAL TRANSMISSION AND FARADAY ROTATION OF A SINGLE G-FET

We model a g-FET by a system, where graphene with optical condictivity $\sigma_\pm$ (for RH and LH circular polarizations respectively) is deposited on a substrate consisting of two layers (1 and 2) characterized by the complex refractive indices $N_1 = n_1 + ik_1$ and $N_2 = n_2 + ik_2$ with the thicknesses d_1 and d_2 respectively (Supplementary Figure 1). In the present case the first layer is SiO_2 with d_1 = 300 nm, while the second layer is Si with a thickness of several hundreds of microns. Due to the very small thickness of the first layer, the transmission is mostly determined by the second layer (except in the optical region where SiO_2 shows a phonon absorption, which is beyond the considered

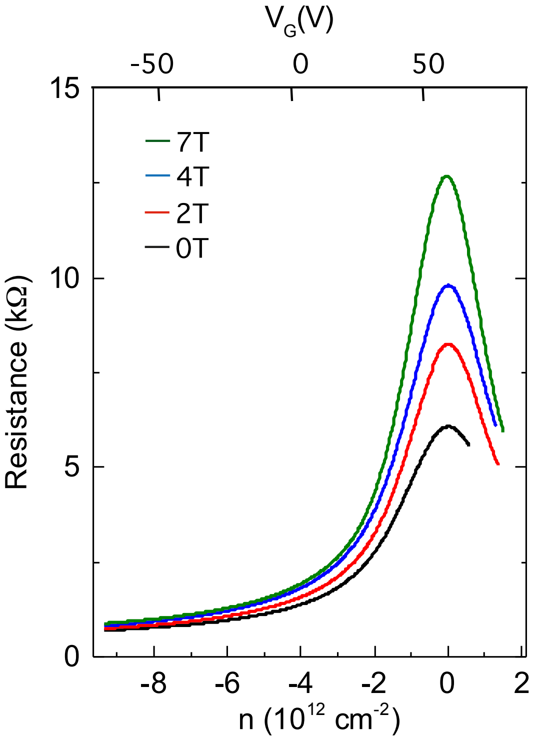

Supplementary Figure 3. Magneto-resistivity curves of a patterned g-FET at 250 K, with the same parameters as in Figure 3 of the main text. They are similar to the transport characteristics of continuous graphene (Figure 1 of the main text).

THz range). Nevertheless, for the sake of accuracy we take both layers into account in our calculations.

First we compute all relevant Fresnel coefficients (defined in the figure): $t^{\pm}_{\mathrm{vg1}} = 2/(N_1+1+Z_0\sigma_\pm)$, $t_{12} = 2N_1/(N_1+N_2)$, $t_{21} = 2N_2/(N_1+N_2)$, $t_{2\mathrm{v}} = 2N_2/(N_2+1)$, $r^{\pm}_{1\mathrm{gv}} = (N_1-1-Z_0\sigma_\pm)/(N_1+1+Z_0\sigma_\pm)$, $r_{12} = (N_1-N_2)/(N_1+N_2)$, $r_{2\mathrm{v}} = (N_2-1)/(N_2+1)$, $r_{21} = (N_2-N_1)/(N_1+N_2)$, $\phi_1 = \exp(i\omega N_1 d_1/c)$ and $\phi_2 = \exp(i\omega N_2 d_2/c)$, where Z_0 is the impedance of vacuum. Note that in the thin-film approximation the effect of graphene can be included into the Fresnel coefficients $t^{\pm}_{\mathrm{vg1}}$ and $r^{\pm}_{1\mathrm{gv}}$.

As the thickness of Si is comparable with or larger than the wavelength, the Fabry-Perot effect is normally produced. However, we suppress the Fabry-Perot effect by reducing the resolution, in order to simplify the data analysis. Therefore we should consider separately the cases of (i) a coherent (amplitude) and (ii) an incoherent (intensity) addition of the multiply-reflected waves in the second layer. Treatment of incoherent addition is described in Supplementary Reference 1.

Coherent addition. In this case, we can calculate the total amplitude transmission coefficients:

$$t_\pm = \frac{t^{\pm}_{\mathrm{vg1}} t_{12} t_{2\mathrm{v}} \phi_1 \phi_2}{1 - r^{\pm}_{1\mathrm{gv}} r_{12}\phi_1^2 - (r_{21} + r^{\pm}_{1\mathrm{gv}}\phi_1^2) r_{2\mathrm{v}}\phi_2^2}. \tag{1}$$

The measured intensity transmission coefficients for RH and LH circular polarizations and the Faraday rotation are then obtained straightforwardly: $T_\pm = |t_\pm|^2$ and $\theta_{\mathrm{F}} = (1/2)\mathrm{Arg}(t_- t_+^*)$.

Incoherent addition. In this case, the phase information in ϕ_2 is lost and intensities are added rather than amplitudes in the second layer. Therefore we have:

$$T_\pm = \frac{\left|t^{\pm}_{\mathrm{vg1}} t_{12} t_{2\mathrm{v}} \phi_1 \phi_2\right|^2}{\left|1 - r^{\pm}_{1\mathrm{gv}} r_{12}\phi_1^2\right|^2 - \left|r_{21} + r^{\pm}_{1\mathrm{gv}}\phi_1^2\right|^2 \left|r_{2\mathrm{v}}\phi_2^2\right|^2}. \tag{2}$$

$$\theta_{\mathrm{F}} = \frac{1}{2}\mathrm{Arg}\left\{\frac{t^{-}_{\mathrm{vg1}} t^{+*}_{\mathrm{vg1}}}{(1 - r^{-}_{1\mathrm{gv}} r_{12}\phi_1^2)(1 - r^{+}_{1\mathrm{gv}} r_{12}\phi_1^2)^* - (r_{21} + r^{-}_{1\mathrm{gv}}\phi_1^2)(r_{21} + r^{+}_{1\mathrm{gv}}\phi_1^2)^* \left|r_{2\mathrm{v}}\phi_2^2\right|^2}\right\}. \tag{3}$$

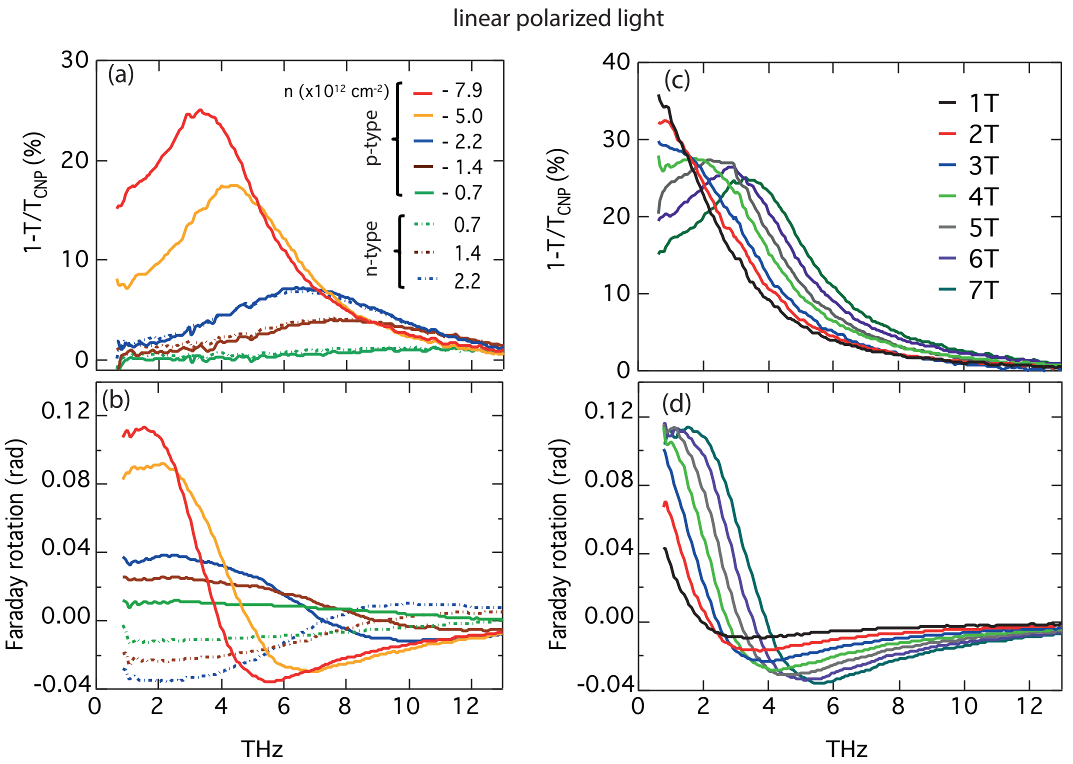

Supplementary Figure 4. The experimental spectra of extinction in linear polarized light and Faraday rotation used to derive the curves on Figures 1c and 1d of the main text. (a) and (b): Linear-polarization extinction (a) and Faraday rotation (b) at B = 7 T for different doping levels. (c) and (d) Linear-polarization extinction (c) and Faraday rotation (d) for $n = -7.9 \times 10^{12}$ cm^{-2} for different values of magnetic field.

Expressions for the reflection coefficients and magneto-optical Kerr rotation angle can be derived in a similar fashion.

II. SUPPLEMENTARY NOTE 2: RELATION BETWEEN TRANSMISSION, REFLECTION, ABSORPTION AND EXTINCTION

A model calculation for a g-FET with a high doping level ($n = -7.2 \times 10^{12}$ cm^{-2}) at 7 T is shown in Supplementary Figure 2. The top panel presents the model reflectivity and transmission spectra for LH polarized light (where the CR is observed) and at a charge neutrality point, where we assume the Drude weight to be zero. In the calculation, the multiple reflections in the substrate were added incoherently, which corresponds to our experiment, where the Fabry-Perot effect was suppressed. One can see that doping has a much stronger effect on transmission than on reflection. The bottom panel compares the absorption $A = 1 - R - T$, which is dominated by graphene charge carriers, and the extinction spectra $1 - T/T_{\rm CNP}$, which can be most accurately measured in the experiment. An important conclusion is that under typical conditions of our experiment the extinction has essentially the same spectral shape as the absorption by the Drude carriers, with a strong peak at the CR frequency ω_c. The difference by about a factor of 2 is due to a high refractive index of Si, which makes the transmission at the CNP close to 50%.

Supplementary References

[1] Harbecke, B Coherent and incoherent reflection and transmission of multilayer structures, *Appl. Phys. B* **39**, 165-170 (1986).
